# Interpreting the Epstein-Barr Virus (EBV) Epigenome Using High-Throughput Data

**DOI:** 10.3390/v5041042

**Published:** 2013-04-02

**Authors:** Aaron Arvey, Italo Tempera, Paul M. Lieberman

**Affiliations:** 1 Memorial Sloan Kettering Cancer Center, NY, NY and Howard Hughes Medical Institute: E-Mail: aarvey@cbio.mskcc.org; 2 The Fels Cancer Institute and Department of Microbiology Temple University School of Medicine, Philadelphia, PA; E-Mail: Tempera@temple.edu; 3 The Wistar Institute Philadelphia, PA 19104; E-Mail: Lieberman@wistar.org

**Keywords:** Epstein-Barr virus, gammaherpesvirus, chromatin, histone modification, CTCF, OriP

## Abstract

The Epstein-Barr virus (EBV) double-stranded DNA genome is subject to extensive epigenetic regulation. Large consortiums and individual labs have generated a vast number of genome-wide data sets on human lymphoblastoid and other cell lines latently infected with EBV. Analysis of these data sets reveals important new information on the properties of the host and viral chromosome structure organization and epigenetic modifications. We discuss the mapping of these data sets and the subsequent insights into the chromatin structure and transcription factor binding patterns on latent EBV genomes. Colocalization of multiple histone modifications and transcription factors at regulatory loci are considered in the context of the biology and regulation of EBV.

## 1. Introduction

Epstein-Barr virus (EBV) is a human gammaherpesvirus that establishes long-term latent infection in B-lymphocytes [2,3]. The latent infection is associated with various B-cell malignancies, including Burkitt’s lymphoma, Hodgkin’s disease and lymphoproliferative diseases, following immunosuppression. EBV infection can efficiently immortalize naive resting B-cells and establish long-term quasi-homogenous lymphoblastoid cell lines (LCLs). In LCLs, the majority of viral genomes adopt a gene expression program, referred to as type III latency, which represents the most permissive form of latent infection [4]. In type III latency, the complete set of viral genes required for B-cell proliferation and survival are expressed, while the viral genes required for lytic replication and virion production are repressed. The viral genomes are maintained as multicopy circular mini-chromosomes that reside in the nuclear compartment. Viral gene expression is regulated by a combination of host and viral regulatory factors, and latent replication is limited to once per cell cycle in concert with host chromosomes [5]. While most cells maintain the viral genome in a type III latent state, a percentage of cells in the population can undergo spontaneous lytic replication, and the extent of this lytic replication depends on the LCL and culture conditions [6]. 

To appreciate the relevance of the EBV epigenome, it is first necessary to highlight some of the major properties of the EBV genome during latency. The type III latency-associated gene expression program in LCLs consists of nine protein coding genes, 21 microRNAs and several non-coding RNAs. The protein coding genes include the Epstein-Barr Nuclear Antigens (EBNAs) EBNA-LP, 1, 2, 3a, 3b and 3c, as well as the Latency Membrane Proteins, LMP1, LMP2a and LMP2b. Two small non-coding RNAs, EBER1 and EBER2, are generated by RNA polymerase III. The miRNAs are generated from two different host transcripts from the BHRF1 or BART regions of the genome [7]. The latent genome is circularized through the joining of the terminal repeats (TRs), which generates the template for the LMP2a and LMP2b transcripts. The viral episome is maintained through the interaction of the EBNA1 proteins with the viral origin of plasmid replication (OriP), which consists of a family of repeats (FR) and a dyad symmetry (DS) element. The FR is required for maintenance through a mechanism that involves tethering to metaphase chromosomes and the DS functions as an efficient origin of bidirectional DNA replication. EBNA1 also binds to the Q promoter (Qp), which functions as an alternative promoter for expressing the EBNA1 transcript only. OriP can also function as an EBNA1-dependent transcriptional enhancer of the C promoter (Cp), which controls the transcription of a large multicistronic transcript encoding the EBNA-LP, -2, -3a, -3b, -3c and -1 genes. LMP1 transcription can initiate from the TR or from regions near the TR, and its poly A site resides in the first intron of the LMP2 transcripts that are transcribed in the opposite orientation from the complementary DNA strand of LMP1. Lytic origins of DNA replication remain mostly inactive in LCLs, but contain promoters for non-coding RNAs and miRNAs that can be generated at high levels during latency. How these genetic elements are coordinately regulated may be partly revealed through analysis of the viral epigenome.

## 2. Assaying the EBV Epigenome

Epigenetically regulated loci in the EBV genome can be elucidated by high-throughput sequencing data in latently infected human cell lines. Large data sets generated by labs around the world are deposited in standardized databases, such as the NCBI sequence read archive (SRA) and gene expression omnibus (GEO). The raw data can be downloaded and reanalyzed with respect to EBV by aligning the reads to EBV and subtracting any reads that map to the human genome [1]. We have developed a simple open access browser for viewing ENCODE ChIP-seq data sets mapped to the EBV genome (http://ebv.wistar.upenn.edu). The data deposited to this site include raw alignments, coverage tracks and use original accessions as filenames to ensure reproducible analysis.

Due to the small size of the EBV genome relative to the human genome, the alignment can be performed orders of magnitude faster by common tools, such as bowtie and bwa [8,9]. Interestingly, the average number of reads mapping to the viral genome tend to be an order of magnitude more than what would be expected from a randomly selected equally sized portion of the human genome given the estimated episome copy number. This suggests that the viral chromatin may be more soluble and/or amenable to sonication and enzyme digestion chromatin fragmentation.

The EBV genome contains several loci that should be interpreted with caution when using sequencing data. Regions that are seemingly depleted may in fact be regions whose copy number was overestimated (and thus, over-normalized, e.g., terminal or W repeats) or has orthologous regions in the human genome and, therefore, is unmappable (e.g., the simple repeat elements in EBNA1 and EBNA2). Furthermore, an initial challenge in any large data study is segregating the data into what is robust, spurious or artifactual. In the case of ChIP, the traditional controls of sonicated genomic DNA (“input”) and non-specific IgG ChIP provide information on two independent background noises. The EBV genome has no regions that appear enriched in the input controls, indicating that the genome is fairly uniform with no genomic regions being more easily sonicated than others. However, the FR repeats are enriched in several IgG ChIP controls, which suggests that FR lacks antibody specificity and is likely to be some form of “sticky” chromatin, possibly due to its potential role as a nuclear matrix attachment region [10]. Even though FR immunoprecipates upon non-specific IgG interrogation, it is possible that this non-specific interaction occurs *in vivo*, with many proteins genuinely binding the chromatin. However, these two scenarios cannot be disambiguated using current technologies.

Since meta-analyzing genomics experiments for EBV comes with the same caveats as analyzing data for human, it is highly recommended that all experiments be first mapped to the human genome. Quality statistics should be generated from the typically millions of mapping reads and thousands of relevant human loci instead of the typically <20 sites in the viral episome. Quality metrics include cross-strand correlation, which is an effective measure for fragment length and enrichment relative to genomic background [11] and enrichment estimates via percentage reads in peaks.

Experiment reproducibility should also be examined in both the human and EBV genomes. However, even examining biological replicates across only the EBV genome can generate an estimate of reproducibility. For instance, NF-kB experiments give widely varying peak results. In this case, only the best replicates can be selected from the human aligned data. In some cases, even when an experiment is highly reproducible in human, a low number of reads mapping to the viral episome makes one or multiple replicates unreliable; however, this has mostly ceased being a problem with the advent of deeper sequencers.

One complication of analyzing the EBV epigenome through publically available data is that most LCLs are transformed using the B95.8 genome, which contains a large deletion in the Bam HI A region that encompasses the duplicated lytic origin (OriLyt right or DSr) and many BamHI A non-coding RNAs and miRNAs [7,12]. Even though the B95.8 strain is more commonly used, all recent studies, including our own, map to the complete EBV reference genome (NC_007605). Consequently, many of the data sets fail to map to the BamHI A region and lack information on the chromatin structure and transcripts generated from this region. Nevertheless, much information exists for the remaining regulatory elements of the B95.8 genome, which functions efficiently in B-cell immortalization and maintenance of latent infection.

## 3. Tour of the EBV Epigenome

### 3.1. Overview of Chromatin Structure

In latently infected cells, the EBV genome is chromatinized with density similar to that of the host genome. Nucleosome position and histone tail modifications are strong indicators of chromatin structure and gene regulation. Micrococcal nuclease I (MNase I) and DNase I mapping studies can be used to assess the overall chromatin density and structure of viral genetic loci. For EBV, the majority of the genome is occupied by nucleosomes with varying degrees of static positioning or phasing, consistent with the dynamic nature of the viral genomes in LCL populations [13]. 

**Figure 1:**
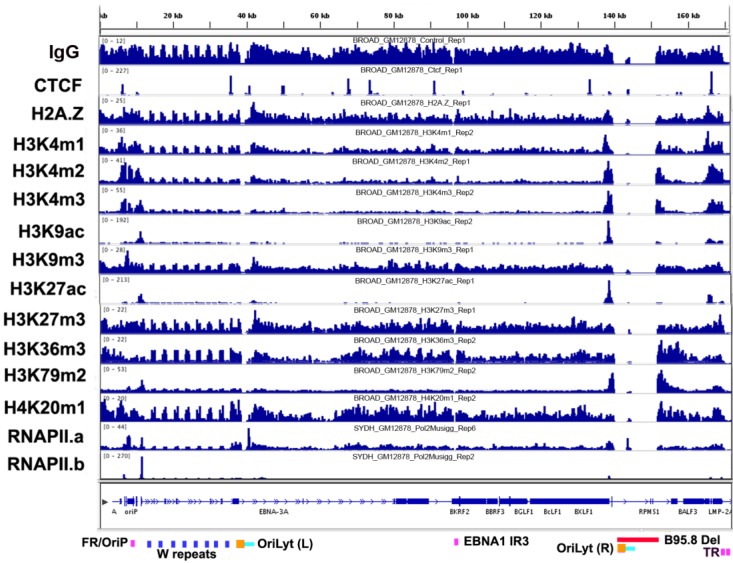
Chromatin overview of the Epstein-Barr virus (EBV) epigenome. ChIP-seq in lymphoblastoid cell lines (LCLs) are mapped to the wild-type HHV4 genome for CTCF, histone variant, H2A.Z, histone modifications and RNAPII. Many colocalized modifications are sites of type III latent gene transcription, such as Cp, BARTp and the LMP locus. RNAPII tracks illustrate the heterogeneous and dynamic nature of recruitment across the viral genome.

### 3.2. Histone Modifications

Mapping histone modifications across the EBV genome reveals enrichment of several marks at specific genome positions (Figure 1B). Enrichment can be assessed based on background input or IgG control DNA, as well as relative to average values for all histone modification specific ChIP assays and MNase I nucleosome density mapping [14]. Using these guidelines, histone modifications, H3K4m1, H3K4m2 and H3K4m3, are similarly enriched at the EBER-OriP-Cp locus (~7,000–13,000), the BART transcript promoter region (BARTp) (~138,500) and the LMP2-LMP1 promoter locus (~165,400–169,600). These regions represent the major sites of RNA polymerase II and III loading for type III latency transcripts. This is consistent with the established role of H3K4 methylation in transcription enhancer and promoter function, as well as sites of DNA replication [15]. Acetylated histones (H3K9ac and H3K27ac) are enriched at much sharper peaks that correlate well with sites of transcription initiation at Cp (~11,537), the BARTp (~138,563), LMP1p (~169,246) and LMP2a promoter region (~165,319).

Histone modifications associated with facultative (H3K27m3) or constitutive (H3K9m3) heterochromatin appear generally low throughout the EBV genome. This may be due to the type III latency program in which most of the latent genome is transcribed. It may also be due to the partial or abortive lytic gene expression observed in some LCLs. In contrast, the latent KSHV genome has several broad peaks of H3K9m3 and bivalent K3K27m3 and H3K4m3 marks at lytic switch regulatory regions [16]. For EBV, only modestly enriched peaks for H3K27 and H3K9 trimethylation and no apparent bivalent control regions exist. The enrichment of H3K9m3 at the FR region of OriP, while very likely to be the result of sticky chromatin, since this locus also IPs with IgG, is potentially intriguing, because of colocalization with the Origin Recognition Complex (ORC), which has been implicated in H3K9 heterochromatin formation, as well as in replication origin function [17,18]. H3K27m3 is modestly elevated at the BHRF1 promoter control region (~41,852), which may regulate aspects of EBV miRNA production. H3K79m2 is found elevated at the 5’ end of the Cp generated EBNA2 transcript (~11,292) and the BARTp generated BART transcripts (~139,054–155,254). H3K79m2 is conferred by the Dot1 methyltransferase, and recent studies have implicated Dot1 and H3K79m2 in pluripotent stem cell reprogramming [19]. Additionally, Dot1 and H3K79m2 have been implicated in controlling DNA damage response during DNA replication and colocalizing with BAT3 transcription factors [20]. Each of these potential functions are worthy of further investigation at EBV regulatory elements.

### 3.3. CTCF and Cohesin Binding Sites

CTCF is an eleven zinc finger DNA binding protein that has been implicated in chromatin boundary function, enhancer blocking and DNA-loop formation [21,22]. CTCF ChIP-seq reveals at least 19 sites of significant enrichment in multiple replicates, each of which contains a strong CTCF binding motif. These include binding sites at ~6,559 (5’ EBER-1 promoter), ~10,494 (5’ of Cp), ~36,000 (5’ EBNA2 ORF), ~40,792 (OriLyt between divergent promoter of BHLF1 and BHRF1), ~49,973 (5’ Qp), ~67,812 (BMRF1 ORF), ~73,845 (BSLF1/BMLF1 ORF), ~91,290 (BZLF1p), 99,028 (BKRF3 ORF); 133,524 (BVRF1 ORF), 139,033 (BART intron), 143,866 (BARF1 transcript) and 166,446 (LMP2 first intron/LMP1 poly A). It is remarkable that most of these CTCF sites can be assigned to important regulatory regions of the genome. However, it is impossible to assign a single function to CTCF that explains the binding to each of these sites. Surprisingly, many CTCF binding sites are proximal to RNA polymerase regulatory elements, which is in contrast to the host genome, where the vast majority of CTCF sites are located at positions far from transcription initiation. This finding is consistent with other gammaherpesvirus studies, including those with Kaposi's sarcoma-associated herpesvirus (KSHV) multicistronic LANA-vCyclin-vFLIP transcript, that suggest CTCF regulates RNA polymerase programming [23,24]. It is also likely that some of these CTCF sites represent DNA loop junctions and inter-chromosomal linkages, as was found for the CTCF-mediated interactions between the OriP and Qp [25] or OriP and LMP1/2 region [1]. It is also worth noting that CTCF peak heights vary substantially, suggesting that some sites may be stronger or perhaps only bound to a subset of episomes.

### 3.4. Transcription Factor Binding Sites

**Figure 2:**
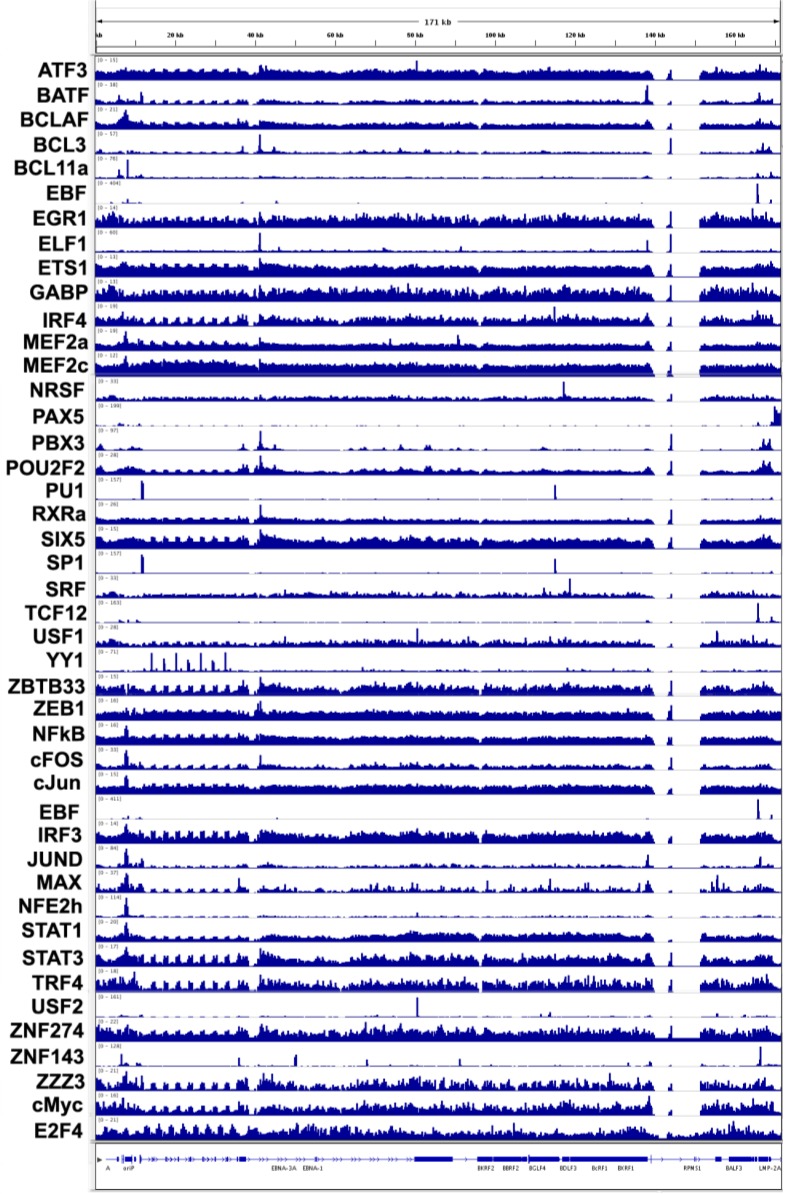
Transcription factor occupancy on the EBV epigenome. ChIP-seq tracks for various transcription factors (as indicated) were mapped from B95-8 LCLs. There is extensive colocalization at multiple loci across the genome.

EBV gene expression is regulated by mechanisms similar, if not identical, to host cell genes. Therefore, it is not surprising that many cellular transcription factors bind at multiple locations across the EBV genome (Figure 2). Transcription factor binding at known viral promoter regulatory elements is expected, and many of these interactions have been described previously. For example, PU.1 and Sp1 co-regulate Cp/Wp and LMP1 and are found colocalized at these loci and at an unanticipated site within a cluster of lytic genes (e.g., BGLF1 ORF) not expressed during latent infection. The function of PU.1/Sp1 binding at this site in latently infected LCLs is not obvious. YY1 has also been implicated in regulation of Wp and is highly enriched in at least one, possibly all, W repeats. Given the role of YY1 in polycomb-mediated chromatin regulation [26], it is tempting to speculate that the function of YY1 in these repeats is related to H3K27m3 formation and higher order chromatin organization at these internal repeats.

Transcription factor co-occupancy is observed at several key regulatory elements of EBV. The combination of factors at each of these sites may provide interesting new insights into signaling pathways and interactive transcription factor networks. For instance, BATF, JunD, Max and TRF4 are enriched at Cp; OriLyt-L (BHLF1/BHRF1 divergent promoter) contains binding sites for BCL3, ELF1, PBX3, POU2F2, RXRa and cFOS; and the LMP2 promoter binds TCF12, EBF, ZNF143 and JUND. Several cellular factors were found to bind to regions of the viral episome with no known regulatory functions. For example, ATF3, USF1 and USF2 show strong colocalization at ~80,655, which falls within the first internal repeat of the EBNA3A transcript. Another example is SRF and NRSF binding at 112,407, which falls near the putative promoter elements of the capsid protein BGLF3 promoter, a lytic protein not likely to be expressed during latent infection. The colocalization of this particular subset of factors at these genetic regulatory elements suggests a partitioning of factor functions and warrants further investigation.

### 3.5. Co-Activator Binding Sites

**Figure 3:**
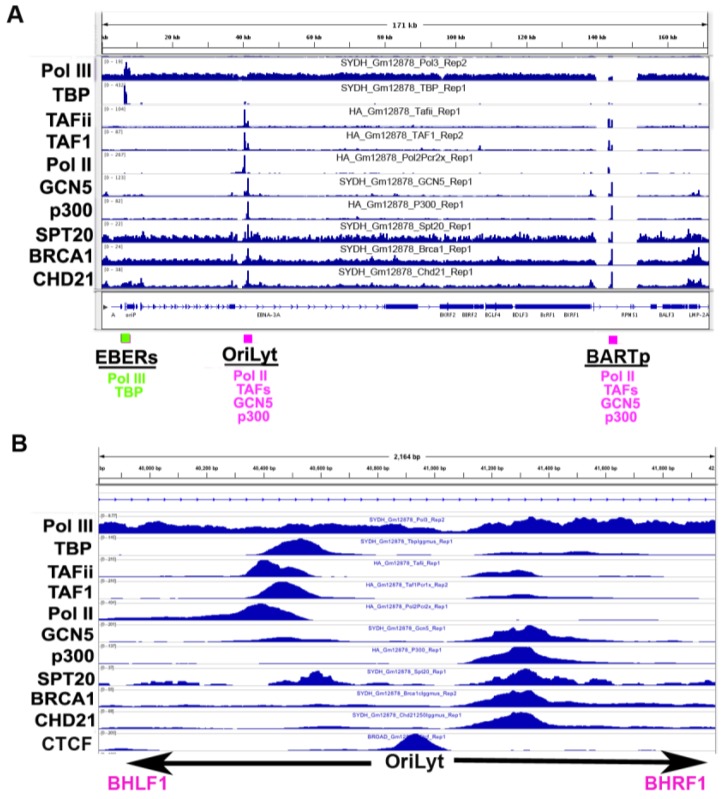
RNA polymerases and transcriptional co-factor occupancy on the EBV genome. ChIP-seq tracks for RNA Pol III, TBP, TAFii, TAF1, RNA Pol II, GCN5, p300, SPT20, BRCA1 and CHD21 for B95-8 LCLs. EBERS oriLyt (R) and BART promoter are indicated below. B) Zoom of the OriLyt region of EBV.

Examination of non-sequence specific transcription co-activators reveals a remarkable enrichment at the OriLyt (L) or OriLyt (R) control elements (Figure 3). In particular, enrichment of GCN5, p300, BRCA1 and CHD21 occurs at the BHRF1 promoter in OriLyt, while TAFs and Pol II are enriched at the divergently transcribed BHLF1 promoter. A CTCF binding site sits between these two different regulatory elements, possibly functioning as a latent/lytic insulator. Also remarkable is that TBP was highly enriched at the EBERs, colocalizing with RNA Pol III.

### 3.6. Origin of Latent Replication

**Figure 4:**
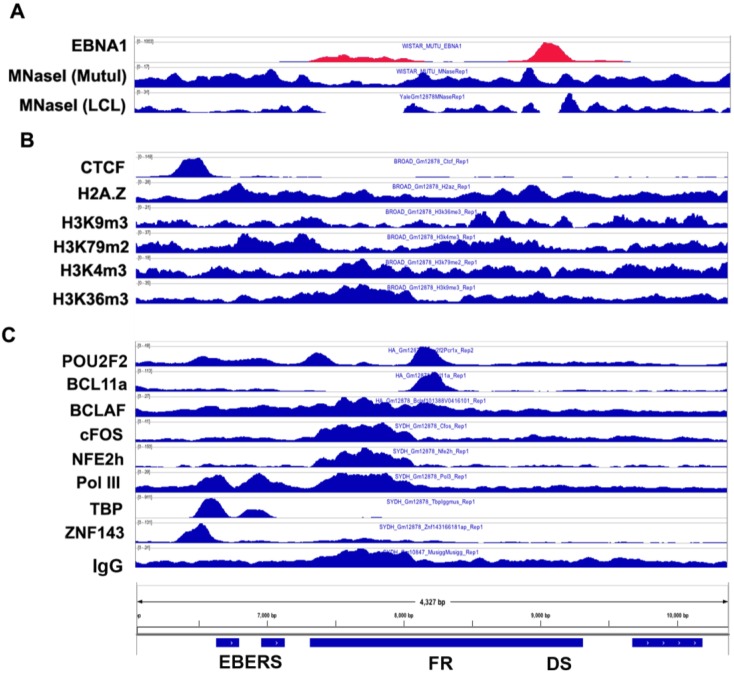
Histone modifications and transcription factor occupancy at OriP. (**A**) MNase I seq analysis for MutuI (type I latent lymphoma cell line) and MutuLCL (type III latent LCL using the same viral strain as MutuI), showing nucleosome depletion at the EBNA1 binding sites in DS. (**B**) ChIP-seq for CTCF, H2A.Z, histone modifications and EBNA1 binding at OriP region. (**C**) ChIP-seq tracks for transcription factors CTCF, POU2F2, BCL11a, BCLAF, cFOS, NFE2h, RNA Pol III, TBP, ZNF143 and EBNA1 at the OriP region.

The origin of latent replication (OriP) is the episomal maintenance element that can serve as an origin of DNA replication and tethers the viral genome to host metaphase chromosomes during mitosis [27,28]. As such, it is possible that some of the factors that associate with OriP may reflect close interactions of OriP with host chromosomal proteins or histone modifications. As mentioned above, OriP can function as a transcriptional enhancer for Cp and LMP1/LMP2, and recent studies have implicated CTCF and cohesins in loop formation between OriP and these promoters [1,25]. CTCF binding sites appear to bracket the entire EBER-OriP region, potentially forming a functional DNA loop for OriP enhancer mobilization and insulation of other gene activation. Examination of the epigenomic features of OriP reveals a nucleosome-free region overlapping EBNA1 binding sites at FR and DS, but a strong positioning and phasing of nucleosomes at positions flanking DS and FR (Figure 4A). Strong nucleosome position flanking DS was reported previously using conventional methods [29]. The epigenetic modification of these nucleosomes are not clear, since H3K4m3 was reported to be elevated, but has significantly higher peaks at regions 5’ to FR and overlapping EBER transcripts. H2A.Z appears to be enriched at these positions, but the relative enrichment is modest. 

As mentioned above, several transcription factors colocalize at OriP. Previous studies have shown that Oct2 (Pou2F2) can bind to FR, and ENCODE ChIP-seq shows strong enrichment of Pou2F2 at FR (Figure 4B). A number of other factors show ChIP-seq signal at FR (e.g., BCLAF, BCL11a, NFE2h, cFos, RNA Pol III and others); however, this region is also elevated in non-specific IgG immunoprecipitation. BCL11a showed a more discrete peak that overlapped with the Oct2 binding site, suggesting that this may reflect-specific binding. As noted above, the relative enrichment of IgG at the FR complicates interpretation of ChIP-Seq data and may reflect important physical features of OriP, including nuclear matrix attachment [10].

### 3.7. DNA Methylation

DNA methylation contributes to the balance of limiting gene expression and avoiding immune detection during latency, while establishing a landscape that can be overcome during lytic reactivation to express the >70 gene products involved in replication. The EBV genome packaged in virions is unmethylated and gradually becomes methylated by host factors during initial cellular infection. Genome-wide analysis of methylated CpG levels in the EBV genome has revealed that the origin of plasmid replication OriP, the Cp and the Qp promoters, and the region for the noncoding RNA EBERS lack significant levels of DNA methylation in lymphoblastoid cell lines during latency [30,31]. Interestingly, CTCF demarcates the boundaries of unmethylated high CpG frequency regions in EBV, and at least in the case of the Qp and Cp, the loss of CTCF binding alters the functionality of these regions [31,32]. Highly methylated loci inhibit the transition from the latent to lytic phase; however, the Zta transcription factor selectively activates methylated promoters of lytic genes, including genes encoding for the viral helicase, the DNA polymerase and the DNA polymerase processivity factor [33,34].

### 3.8. Negative Results

Through meta-analysis, it is possible to discover and observe many phenomena; however, just as notable are the phenomena that are not observed. While negative results are typically not formally reported, they can be accrued in unbiased databases of experiments mapped to the viral genome [1]. For instance, of the 68 transcriptional regulators previously examined, only 26 have had reproducible binding sites in the EBV genome. While some of the 42 TFs may bind the viral genome, possibly with lower affinity, it seems more likely that the majority of host transcriptional regulators do not physically interact with the viral episome. Furthermore, repressive histone modifications, such as H3K27me3, are largely absent from the viral genome (or at the very least, had no spatial enrichment, meaning that if H3K27me3 is present on viral nucleosomes, the modification lacks spatial regulatory specificity).

Previously identified regulatory interactions are largely confirmed in high throughput analyses. However, a small subset of experiments yielded surprising negative results. For instance, NF-kB binding at LMP1p was either weak or non-existent. Additionally, ZEB1 binding to Zp could not be confirmed. In both of these examples, it was crucial that positive controls in the viral and host genomes were provided. Importantly, the ChIP-seq methods are only semi-quantitative, and observed peaks of significant interest need to be validated by conventional ChIP and qPCR methods, which have been performed at only a small subset of these sites.

## 4. Conclusions

The EBV epigenome, as revealed by data mining, reflects only a small fraction of the protein interactions and histone modifications that define the viral chromosome. It is certainly not a complete nor comprehensive characterization of the proteins and modifications that regulate the EBV genome in all its dynamic complexity. Nevertheless, the insights gained from this “tip of the iceberg” glimpse of the EBV epigenome suggest that this discovery approach can reveal many new and previously unanticipated regulatory features of viral-host interactions, gene regulation and chromosome organization. Many of the observations discussed in this review need to be experimentally validated and further characterized to fully assess their functional significance. However, these observations indicate that “omics” dissection of viral and host gene regulation can generate new concepts and hypotheses and a deeper understanding of how the viral and cellular genomes persist during latent infection.
